# Gaming Platforms for People with ASD

**DOI:** 10.3390/jintelligence12120122

**Published:** 2024-11-27

**Authors:** Irini Chaidi, Pantelis Pergantis, Athanasios Drigas, Charalampos Karagiannidis

**Affiliations:** 1 Net Media Lab & Mind & Brain R&D, Institute of Informatics & Telecommunications, National Centre of Scientific Research ‘Demokritos’ Athens, 15341 Agia Paraskevi, Greece; 2 Department of Special Education, University of Thessaly, 38221 Volos, Greece; karagian@uth.gr; 3 Department of Information & Communication Systems Engineering, University of the Aegean, 83200 Karlovasi, Greece

**Keywords:** serious games (SGs), autism spectrum disorders (ASD), platforms

## Abstract

Autism spectrum disorder (ASD) has a significant impact on a person’s social, emotional, and communication functioning. According to research, individualized instruction can significantly improve these deficits. One of the most successful methods of achieving this outcome is by gaming platforms that provide serious games (SGs). This article is a systematic review study using the PRISMA diagram that delves into current research on the characteristics and design criteria of serious gaming platforms suitable for people with autism, presenting the benefits of using serious gaming platforms and highlighting the importance of differentiated strategy and planning, as well as disadvantages such as financial cost and complexity. According to the conclusions of this analysis, the bulk of these programs focus on prototyping and strengthening social and emotional abilities. It is also emphasized that platforms aiming at a bigger audience of persons with ASD, as well as a larger sample size, are required.

## 1. Introduction

Deficits in social interaction, communication disorders, and the emergence of restricted and repetitive patterns of behavior are hallmarks of autism spectrum disorder (ASD). Individuals with ASD may experience a variety of symptoms, such as social difficulties, communication difficulties, and repetitive behaviors (Konstantinidis et al. 2009). Individuals with ASD present challenges in understanding social cues, forming and maintaining friendships, and understanding the emotions of others. In terms of communication, they have problems understanding language, expressing emotions, and communicating effectively. In addition, they often exhibit repetitive and stereotyped behaviors, such as persistence in certain activities and resistance to change. ASD, which is not categorized as a medical condition, is believed to result from a mixture of genetic and environmental influences. Genetic variations and certain environmental exposures during pregnancy are thought to contribute to the development of autism. Diagnosing autism involves a comprehensive approach. Observations of behavior and interactions are combined with various tests, including developmental assessments and hearing tests, to ensure an accurate diagnosis (Konstantinidis et al. 2009). Although there is no cure for autism, there are a variety of treatments aimed at enhancing skills and improving the quality of life of people with ASD. Behavioral therapy focuses on modifying behavior by helping individuals gain social, communication, and self-care skills. Special education tailors learning to individual needs, allowing students to progress at their own pace (Baron-Cohen and Bolton 2010). Drug therapy may be used to treat specific symptoms, usually in combination with other treatments. Support for people with autism is vital to their well-being. This support can be provided from a variety of sources, including family, friends, community organizations, and government programs (Hayes et al. 2010).

Research indicates that positive outcomes are possible for individuals with ASD if appropriate education and intervention are provided. Optimistic approaches include the inclusion of serious games designed for purposes other than entertainment. Serious gaming is highly effective in improving social and communication skills in people with ASD (Chaidi and Drigas 2023). The use of digital games in the educational process encourages interactivity and de-escalation of tensions, as well as active educational models and new opportunities for communication, collaboration, and learning, allowing the student with autism to be introduced in a controlled, predictable, and free setting. Social cues are used to regulate an individual’s attention and focus during an activity (Chaidi and Drigas 2023).

SGs are a type of digital game that has witnessed significant growth in recent years. There are multiple interpretations of serious games, such as “a serious game is defined as an intellectual competition, played with a computer according to specific rules, that uses entertainment to promote governmental or corporate, educational, health, public policy, and strategic communication goals” (Zyda 2005). “Serious games” are described as digital games and equipment intended for educational purposes rather than amusement (Sorensen and Meyer 2007). Serious games include simple digital games, mixed art, storytelling, and programming (Zyda 2005). They differ in that they feature educational activities meant to teach, transfer information, and encourage the development of diverse abilities, employing principles of entertainment, creativity, and technology to construct the game to serve certain aims such as issue resolution, which is the core goal of serious design (Belanich et al. 2004). The authors of (Abt 1970) recognized that the capacity to offer direct feedback to students about their performance is crucial and should be supplied according to students’ requirements, which is a benefit of utilizing serious games for teaching since they provide rapid and timely feedback. Serious games, designed for purposes beyond entertainment, have a rich history and versatile applications in education, training, and healthcare. The beginning of serious games can be traced back to the early 1970s when the US military incorporated games into soldier training. After the transition to the 1980s, serious games found a foothold in education, with titles such as “The Oregon Trail” and “Where in the World is Carmen Sandiego?”. The 1990s then witnessed their foray into healthcare with games such as “Virtual Hospital” and “Body Works” (Connolly et al. 2012).

These serious games include several types, each serving a distinct purpose. (A) While serious educational games concentrate on coaching particular tasks or skills, serve a wider range of purposes, including training and awareness, and frequently incorporate educational features (but are not limited to them), educational games are explicitly focused on learning objectives and aim to impart new skills or knowledge. (B) Healthcare games help promote health and manage medical conditions, and social impact games address broader social issues such as poverty, climate change, and discrimination (Kapp 2012). The advantages of serious games over traditional educational methods are remarkable. They offer enhanced engagement and motivation, providing immersive and interactive learning experiences. Their adaptability to individual needs, combined with the ability to measure progress and offer feedback, enhances their effectiveness. Research supports the effectiveness of serious games, demonstrating their ability to teach a wide range of skills, from academic subjects such as math and science to practical tasks such as flying airplanes and operating machinery (Deterding et al. 2011). Looking ahead, the future of serious gaming looks bright. Continuous technological developments are expected to further enhance their immersive qualities. In addition, serious games are likely to expand their applications, addressing social issues and promoting lifelong learning. As they continue to evolve, serious games will play an increasingly integral role in various fields (Garris et al. 2002).

Serious games, designed for purposes beyond entertainment, exhibit distinct characteristics that contribute to their effectiveness in education, training, and healthcare.

- Immersiveness: One of the paramount features of serious games is their immersiveness. Leveraging techniques such as 3D graphics, virtual reality, and sound design, these games draw players into a compelling game world, creating a profound sense of presence (Garris et al. 2002).
- Interactivity: Serious games prioritize interactivity, granting players control over the game world and influencing the game’s outcome. This interactive element empowers players to learn and explore at their own pace, aligning with their interests (Kapp 2012).
- Feedback: Integral to serious games is the provision of feedback to players. Whether conveyed through in-game messages, scores, or rankings, feedback serves as a valuable tool for players to enhance their skills and deepen their understanding (Deterding et al. 2011).
- Challenge: Maintaining an optimal balance between engagement and frustration, serious games should present an appropriate level of challenge. Tailoring the challenge to individual player needs ensures an experience that is both stimulating and attainable (Kapp 2012).
- Motivation: Serious games employ various techniques to foster player motivation. Elements such as competition, rewards, and progression contribute to sustaining players’ interest and commitment to the game (Garris et al. 2002).
- Learning: At the core of serious games is their capacity to promote learning. Techniques such as gamification, simulation, and problem-based learning are employed to create an environment conducive to educational objectives. As players navigate challenges, they acquire knowledge and skills dynamically and engagingly (Deterding et al. 2011).
- Clarifying the distinctions between a serious game and a platform, a serious game and a video game, and a serious game and a website that features games would be beneficial.

### 1.1. Difference Between a Platform and a Serious Game

One of the essential components of serious games is a platform, which consists of both hardware and software components. This platform gives developers access to essential tools and features that enable them to create novel and creative experiences, creatively express their ideas, and transform their ideas into virtual reality in a flexible setting (Wahlman 2017).

It is crucial to remember that serious games are not just enjoyable for those with ASD; they are also a well-designed, multifaceted creation that allows players to develop and try a variety of experiences in addition to having fun. Since serious gaming makes use of the platform’s full ability to provide experiences that surpass the typical goals of entertainment, its ultimate objective transcends simple amusement.

The key distinction is that a platform acts as a flexible tool that enables creators to create serious games that are suited to particular aims and objectives (Pavkov et al. 2017). 

The mutually beneficial relationship between platforms and serious games highlights the platform’s significance as a catalyst for creativity and innovation. Using the platform’s integrated tools and capabilities, developers can freely express their creativity and produce thrilling serious games with a specific goal in mind (Wahlman 2017).

### 1.2. Difference Between a Serious Game and a Video Game

A video game is an interactive electronic game that can be played on a computer, mobile device, or video game console. These games can be used for a variety of objectives, including training and teaching, although their primary function is enjoyment (Patti 2022).

A serious game, on the other hand, is a specific type of game that was created with a purpose other than amusement. Applications of serious games in training, education, healthcare, and social effects are only a few of its many uses (Hookham et al. 2016).

The primary difference is seen in the reason behind the creation of these games. Serious games are purposefully made to accomplish particular goals, such as teaching people new skills or getting them ready for particular jobs. They are effective resources for skill development and focused learning (Patti 2022).

On the other hand, the primary goal of video games as a whole is amusement. Although they might provide captivating and immersive experiences, their primary goal is relaxation and enjoyment rather than achieving a particular training or educational goal. Video games’ dual nature, which straddles both entertainment and education, highlights the wide range of interactive digital experiences available (Patti 2022).

Unlike serious games, which are classified by goal and include simulation, educational, advergame, political, and evangelistic games, video games are classified by gameplay (Putnam and Chong 2008).

### 1.3. Difference Between a Serious Game and a Website with Games

In essence, a website with games is an online platform that offers a range of games for various uses, such as training, education, or amusement. A serious game, on the other hand, is a unique genre created especially with a purpose other than amusement in mind. Serious games are purposefully created to accomplish predetermined goals, such as teaching new skills or offering training for particular work tasks, even if they can be distributed through online channels (Garris et al. 2002).

A serious game and a website with games differ fundamentally in what they are meant to accomplish. Website games are typically designed to provide users with a source of enjoyment and pleasure. Serious games, on the other hand, are carefully planned with a specific objective in mind, emphasizing the achievement of quantifiable results rather than just fun (Hookham et al. 2016; Pavkov et al. 2017).

From casual enjoyment on gaming websites to purpose-driven serious games, this contradiction illustrates the diverse landscape of digital gaming experiences (Pavkov et al. 2017).

The purpose of this systematic review is to provide a comprehensive review of reliable gaming platforms designed specifically for people with ASD. Through a comprehensive analysis of the current literature, this article aims to provide valuable insights into the key features and requirements for developing effective serious gaming platforms to meet the unique needs of individuals with autism.

## 2. Materials and Methods

### Study Design/Eligibility Criteria

This systematic review study aims to gather recent data on the features of gaming platforms for people with ASD, how they are designed, and if they respond to the specificities of people with ASD.

The present research, through the literature review, attempts to answer the following research questions.

- RQ1. What are the design features of the platforms?
- RQ2. What is the purpose of existing platforms? What educational skills do serious game platforms improve? A critical evaluation of existing platforms aimed at developing and improving the skills of people with ASD.

The inclusion criteria for the main part were the following:

- IC1. The research is addressed exclusively to people with ASD (low- and high-functioning).
- IC2. Consider only platforms with serious games and not digital games in general.
- IC3. Research on gaming platforms as well as their development and design.
- IC4. Research articles dating within the last 10 years from 2014 to the present and written to ensure the objectivity and validity of the information to be provided.
- IC5. Articles written in the English language.

Therefore, the exclusion criteria were as follows:

- EC1. The research is NOT addressed exclusively to people with ASD (low- and high-functioning).
- EC2. Articles referring to games but not for people with ASD.
- EC3. Articles that refer to people with ASD but not serious gaming.
- EC4. Research articles before 2014.
- EC5. Articles not written in English.

The use of these search terms enabled a thorough exploration of the current literature on serious games, covering aspects related to platforms, development methodologies, and design principles. Databases provided insights from the academic community and contributed a wide range of scientific articles offering a unique perspective on serious games in healthcare.

This systematic and multi-based approach aimed to capture a comprehensive view of recent developments and trends in the field of serious gaming. By limiting the search to the past ten years, the intent was to prioritize contemporary research, recognizing the dynamic nature of the serious gaming landscape. The selected articles are expected to contribute valuable and up-to-date information to the understanding of serious game platform development and design.

**Figure 1:**
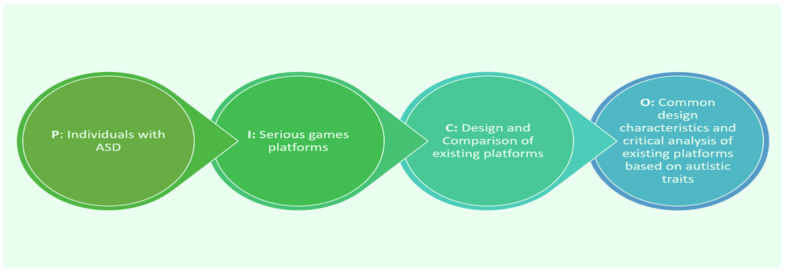
PICO process.

**Figure 2:**
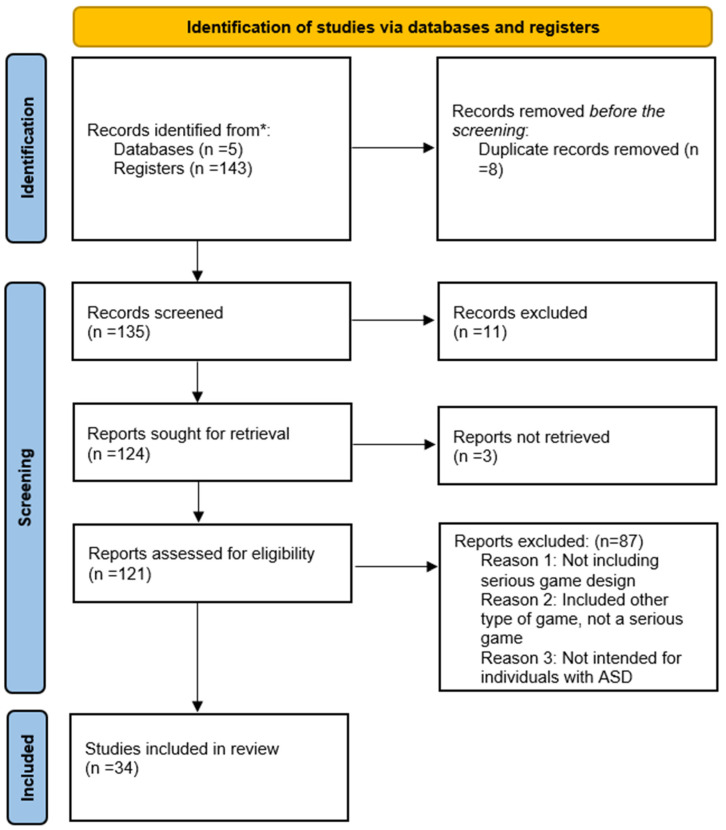
The PRISMA flow diagram (Page et al. 2021).

The research questions were established with PICO (Figure 1). To systematically gather articles for this review, PRISMA 2020 (Figure 2) was used in five prominent academic databases, PubMed, Scopus, Web of Science, Medline, and complementary Google Scholar, using a specific and comprehensive strategy search. The specified search keywords used were “Gamified Environments”, “Serious Games”, “Serious game platform”, “Autistic Spectrum Disorder”, “Revision of research”, “Serious game development”, and “Serious game design”.

According to the selection criteria used in this literature review, 143 articles were identified and screened for the final selection of the included articles, which concluded after processing and inclusion, as well as representative criteria for the main part (*n* = 34) in the final selection for further investigation and analysis. The analyzed literature was from between 2014 and March 2024. The eligibility criteria were created as the first step in the selection process. In addition, a list of keywords was generated to begin the search in the databases, which employed Boolean operators and other search filters to provide the most results. After deleting duplicate research (*n* = 8), the selection was made based on eligibility criteria (*n* = 135) via title and abstract screening. Following the elimination of (*n* = 11) articles, the method proceeded to full-text screening. The remaining research (124 in all) was thoroughly reviewed. Unfortunately, three articles could not be downloaded for full-text screening. The final selection process involved two independent reviewers who analyzed the complete text of *n* = 121 publications and discussed the application of the qualifying criteria. The remaining (*n* = 87) items were eliminated because they did not match the qualifying requirements (did not include serious game design, includes other types of games that are not serious games, or not intended for people with ASD). This leaves a total of 34 items in the final list. Figure 1 and Figure 2 present a summary of the results.

## 3. Theoretical Knowledge

### 3.1. Serious Games and Autism

The authors of Chaidi and Drigas (2023) reported that, in a systematic review of selected studies by Noor et al. (2012) on the use of digital serious games for people with ASD, the term “serious games” is limited to digital games that attempt to improve skills or knowledge beyond pure entertainment, with the term “serious” assigning meaning to “products” in education. The research determined the following classifications: serious autistic games include online computer games, virtual reality, mobile devices, touch screen and desktop games, and interactive games.

Serious games are entertaining and captivating, but their main purpose is to educate, research, and promote, according to Noor et al. (2012). Because of this, the game intentionally sacrifices fun and enjoyment in order to let the player move forward as they choose.

“Serious games can be classified into several types” based on the reason behind their creation. (a) Edutainment: fusing education with enjoyment; (b) Game-based learning, also known as “Game Learning: Educational and Training Games”; (c) Simulation games: games that teach acceptable conduct in the context of simulated situations or conditions; (d) Health games: these include games for cognitive training, physical rehabilitation, and mental health therapy; (e) Exercise games: these are games for fitness; gamification is the process of combining game design and engineering to solve problems and engage audiences. (f) Art Games: video games are made to express artistic ideas or creative notions. (g) Productivity Games: games that provide points for finishing actual activities using to-do lists. Advergames are games that are used to promote products. Finally, Alvarez and Rampnoux (2007) tried to classify serious games into five main categories: advergaming, edutainment, edu-market, diversion, and simulation (Stack 2005).

### 3.2. Serious Games for ASD

It is true that serious games are frequently employed in ASD for three primary reasons:

1. Educational Goals: enhance the cognitive and social abilities of children and adults with ASD, practice commonplace skills such as communication and social situation comprehension, and teach fundamental ideas and abilities such as emotion recognition, problem-solving, and decision-making through interactive settings.
2. Therapeutic Purposes: used to improve behavior and self-regulation, promote emotional understanding, assist people with ASD in identifying and controlling their emotions, teach relaxation and stress-reduction techniques through realistic scenarios, and support certain therapeutic interventions, such as occupational therapy or speech therapy, in conjunction with conventional methods.
3. Other Purposes (socialization, adaptation, and autonomy): allows individuals with ASD to practice skills they will use in real life by fostering a sense of self-confidence and autonomy, provides a safe and controlled environment to try new situations without the stress of direct social interactions, and helps them become accustomed to real situations and develop independence, such as in social interactions and preparing for work.

Serious games are a useful educational and therapeutic tool for individuals with ASD because of these objectives, which help them integrate socially and emotionally and build useful self-care skills.

Until recently, serious games for autism have been designed for two purposes: treatment and education, learning, and training. They are divided into two broad categories according to their purpose. (a) Serious games for education are intended to assist the instructor or student throughout the teaching and/or learning process in understanding money, developing social and communication skills, learning first aid, and storytelling. (b) Therapeutic games are designed to improve visual motor coordination, social skills, sensory integration, electroencephalography (EEG), and social behaviors (Chaidi and Drigas 2023).

A serious game is one that is primarily made for objectives other than simple amusement, such as skill development, education, or training. Serious games are a creative way to get students interested in learning because they use storytelling and interaction to make difficult subjects approachable and interactive.

The following are some ways that serious games support education:

4. Engagement and motivation: Serious games make learning more fascinating and engaging by using the intrinsic appeal of games, including challenge, competitiveness, and achievement (Malinverni et al. 2017). Given that games provide a dynamic and immersive experience, this is particularly crucial for students who might find it difficult to learn using traditional techniques. Additionally, by including feedback, levels, and incentives, games provide students with specific objectives to work toward, enhancing the learning process and promoting sustained focus.
5. Active Learning and Knowledge Retention: Instead of merely allowing students to passively absorb information, games encourage active learning, in which they actively participate and experiment with their knowledge in real-time. Because students are more likely to retain material when they actively apply it, this hands-on approach can boost retention.
6. Simulations and real-world skills: Serious games are helpful for difficult or high-learning scenarios because they frequently incorporate simulations of real-world settings (Mota et al. 2020). Through simulations, students can rehearse situations that would otherwise be too risky, costly, or impractical to replicate in a classroom.
7. Problem-solving and critical thinking: Since serious games promote deeper cognitive processing and develop transferable skills beyond the game’s specific content, they are made to test players’ problem-solving and critical thinking abilities. This encourages them to analyze situations, test hypotheses, and make strategic decisions that aid in achieving educational goals.
8. Instant feedback and flexibility: In order for learning to be effective, serious games must offer instant feedback and adaptability. Because they can see the results of their decisions right away, students are able to adjust and try different strategies. As students learn from their failures and accomplishments in real-time, immediate feedback accelerates the learning process. Furthermore, several games provide a customized learning experience by adjusting to the student’s ability level and learning speed. By providing challenges without leading to irritation or boredom, adaptive learning games can assist students in maintaining their optimal learning state.
9. Cooperation and Social abilities: Many serious games have cooperative or multiplayer components, which motivate students to work together, communicate, and come up with solutions. This helps them build their social and teamwork abilities (Arzone et al. 2020; Derks et al. 2022).
10. Assessment and Data Collection: Because serious games gather information about players’ choices, development, and areas of difficulty, they can also be effective assessment tools. Teachers can utilize this information to gauge students’ comprehension and modify the curriculum to meet their requirements (Goswami et al. 2021; Lu et al. 2022). Compared to typical assessments, continuous assessment provides a more thorough knowledge of students’ talents since it captures their creativity, resilience, and problem-solving techniques.

Serious games for ASD are also utilized in medicine, providing assistance in areas such as evaluation, therapy, and the teaching of health management.

The primary therapeutic benefits of serious games for those with ASD include:

11. Behavioral Assessment and Diagnosis: Real-time behavior monitoring and recording through serious games provides a more realistic view of the responses and abilities of individuals with ASD. Professionals can evaluate memory, attention, problem-solving, and other cognitive abilities using the data gathered, potentially detecting developmental or cognitive deficiencies.
12. Improving Therapeutic Participation: By transforming tasks into engaging and entertaining experiences, serious games help individuals with ASD participate more actively in therapies such as occupational therapy and speech therapy. Through the use of games, therapeutic activities become more accessible and less stressful for those with ASD.
13. Management and Education in Self-Care and Autonomy: Serious games encourage learning fundamental health management and self-care skills, such as adhering to a schedule and comprehending basic medical instructions. People can learn how to identify psychological or physical problems and get help when necessary by playing specially made games (Constain et al. 2019; Ntalindwa et al. 2022).
14. Tracking Development and Customizing Care: Therapists can track the development of people with ASD and adjust interventions to meet their requirements using serious games. Play-related data can be used by medical practitioners to customize treatment plans.
15. Psychological Support and Stress Management: Some serious games teach relaxation and self-regulation techniques, helping people with ASD manage their stress and emotional reactions. They provide strategies to help the individual recognize early signs of stress and use coping techniques (Carlier et al. 2020).

For individuals with ASD, using serious games for medical purposes offers a safe, quantifiable, and customized approach to health and psychological well-being, enhancing the therapy process and bolstering personal autonomy.

## 4. Results

### 4.1. Participants and Study Characteristics

After conducting the research search following the pre-defined selection criteria, a total of 29 studies were gathered and subjected to comprehensive analysis focused on two main aspects: (a) 12 platform design and (b) 17 analysis of existing serious platforms.

A thorough evaluation of the studies allowed data extraction regarding authorship, nationality, period of publication, and study design, which included the method, main aims and procedures, and the target population.

According to the included studies, we collected data from *n* = 29 studies from 2014 to 2024. The research protocols of the included studies were distributed across many countries, including Australia: *n* = 2 ([31,32]), Bangladesh: *n* = 2 ([33,34]), Brazil: *n* = 5 ([35–39]), Canada: *n* = 1 ([40]), China: *n* = 1 ([41]), Greece: *n* = 1 ([42]), Germany: *n* = 2 ([43,44]), India: *n* = 1 ([45]), Indonesia: *n* = 1 ([46]), Italy: *n* = 2 ([47,48]), Malaysia: *n* = 3 ([49,50,25]), Mexico: *n* = 1 ([51]), Peru: *n* = 1 ([52]), Portugal: *n* = 1 ([53]), Saudi Arabia: *n* = 2 ([54,55]), Serbia: *n* = 1 ([56]), Spain: *n* = 2 ([57,58]), Taiwan: *n* = 1 ([59]), the USA: *n* = 3 ([60,61,62]), and the UK & ISRAEL & SWEDEN: *n* = 1 ([63]).

**Figure 3:**
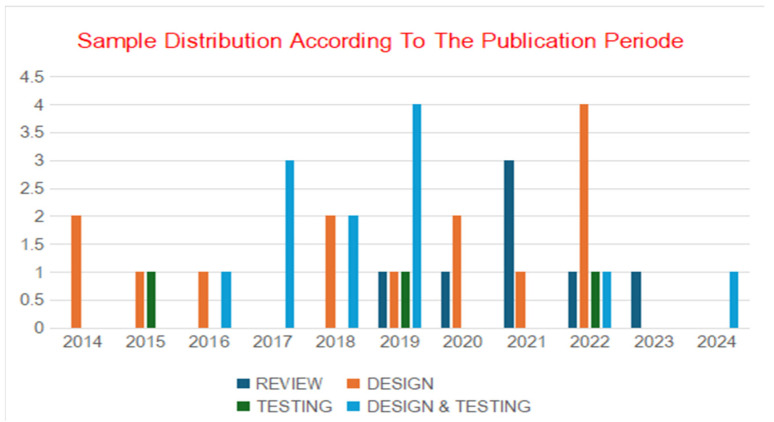
Sample distribution based on the publishing era. Source: Personal explanation.

The results of the research were categorized according to the year of publication. Figure 3 captures the frequency of research publications during the decade of 2014–2024.

**Figure 4:**
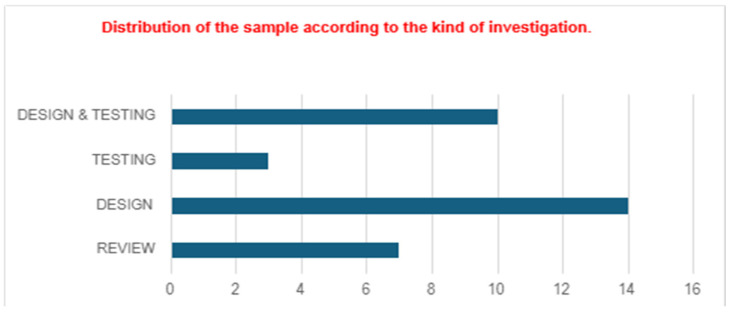
Distribution of the sample according to the object of study. Personal explanation.

In addition, studies were grouped into four study methods, which were the object of study: (a) Literature Review, (b) Design, (c) Testing, and (d) Design and Testing (Figure 4).

### 4.2. RQ1: What Are the Design Features of the Platforms?

For this work, the collected articles were subjected to a comprehensive analysis and focused on platform design.

Platform design: The selected articles were scrutinized to extract information about the features and tools offered by serious gaming platforms. This included a detailed examination of the design elements, functions, and capabilities built into these platforms. By analyzing aspects of platform design, the analysis aimed to provide a nuanced understanding of how serious games are structured and the technological elements that contribute to their effectiveness for people with ASD.

Table 1 summarizes the research that met the inclusion criteria and reported on the design of SG platforms for individuals with ASD.

The systematic review of 30 articles by Camargo et al. (2019) attempts to illustrate a wealth of autism game and user interface design, as well as methodologies and approaches to improve accessibility and aid decision-making in autism software development. While the integration of game elements is generally favorable, defining the target audience and conducting testing may impose additional obstacles in the development process. The review by Whyte et al. (2014) reviews studies aimed at educators, professionals, and parents of people with autism, studies that have tested play-based approaches to improving the lives of autistic children, adolescents, and adults. Other research concentrates on highlighting and defining crucial elements required by designers and practitioners seeking to develop an SG, and the authors of Khowaja and Salim (2014) propose seven components for frameworks. These components include instructional material, learning exercises, user profiles and achievements, game features, game genres, and game mechanics. As well as the fundamental ideas of creating serious computer games, Whyte et al. (2014) applies these findings to therapies for persons with ASD to increase their willingness to play. These factors include (a) fascinating stories that improve motivation and provide context, learning (b) objectives focused on specific skills, prizes, and feedback on goal success (Reward Learning), (c) increasing levels of difficulty and individualized training, and (d) providing options (Baranowski et al. 2008; Kapp 2012). Autism should prioritize four key aspects of serious game design: (1) incorporating story and target behaviors, (2) using cooperative multiplayer games based on the effectiveness of interpersonal interactions in previous virtual interventions in reality, (3) increasing the use of game elements that help in the transfer of knowledge and skills from intervention to real-world situations, and (4) mixed types of schooling and educational support.

As a result, they offer strategies for creating and developing educational games for teachers that take into account both design and instructional factors, as well as the distribution of duties among the participants in the process. A co-design approach is proposed by Tuli and Mantri (2018) for the creation of serious educational games that incorporate educators’ natural participation. The process of idea development involves visualizing the game’s theme or idea. Design, on the other hand, includes creating the user interface, 3D models, animations, and other project-specific elements. Implementation entails developing the codes and algorithms that make the game function. Testing and development also entail analyzing the game to find any bugs or errors before developing the final version. In addition, Bono et al. (2016) developed an automated serious gaming platform to intensively intervene in two core abilities of ASD: imitation and joint attention (JA). This strategy allows for home training. An automated platform for serious gaming allows for extensive mobile intervention. The development of the settings involved mapping two critical skills related to autism spectrum disorder: joint attention (JA) and imitation. Eleven games were made from the Early Start Model Denver: seven for imitation and four for JA. The games featured a wide range of tasks and behaviors including imitation and judgment call (JC), as well as the application of visual and auditory inputs at different levels of complexity. Through the use of a set of automatically extracted quantitative performance measures, the platform, which is mobile device-based, allows the therapist to (1) characterize the child’s initial strengths and difficulties and ensure that the intervention is tailored and appropriate by choosing toys and (2) look into and track the child’s progress over time. Depending on the child’s progress, the therapist can adjust the game or the game’s difficulty levels during the intervention thanks to the platform. Parents and children took part in the sessions at home and at the hospital. All the children participated in every game, but because each game has a different set of rules and each child has a different profile and set of skills, the amount of time spent on each game varies and can be changed by automatic grading. Parents are associated with improving their child’s focus, adaptability, and self-worth as well as the parent–child bond. The viability of employing the created gaming platform for intense at-home intervention is demonstrated by this pilot study.

It is commonly known that fundamental emotion recognition and sensory processing are problematic for those with autism. Thus, in an effort to assist children with autism in overcoming scholastic challenges, researchers are creating adorable and entertaining interactive items. According to an ethnographic study conducted in an autistic school (Wang et al. 2019), teachers prefer using digital technology to supplement traditional teaching strategies and manage behavior, particularly when using serious games. Links between autistic teaching tactics and game design, particularly in the context of mechanics, dynamics, and aesthetics (MDA), were found by examining how teaching strategies work as part of educational activities, including games. In Hunicke et al. (2004), the authors create and evaluate games with three levels. A game’s mechanics are its fundamental elements, such as rules, resources, objectives, and user-controllable options. Behaviors resulting from the combination of player input with game mechanics during gameplay are known as game dynamics. The player’s subjective experience, as well as the feelings and emotions the game aims to evoke, are all considered aspects of a game’s aesthetics. Thus, when creating educational games for children with autism, all three levels should consider the goals of the curriculum and the IEP, which means that individualized content should be included. As a result, a serious game was developed with a design focused on emotion detection skills, with the aim of improving autism education to help children with autism cope with sensory issues at school.

In order to motivate people, researchers Tang et al. (2019) developed a serious game that emphasizes emotion-detecting abilities. The aim of this study was to determine, from the viewpoints of eleven young people with ASD and eleven seasoned specialists, the critical learning and motivational components for serious games meant to improve emotion recognition skills. The goal of this study’s consultative methodology is to gather design recommendations from experts and young people with ASD. The educational and motivational ideas for a serious game are defined in design proposals by adhering to the five serious game principles outlined by Whyte et al. (2014). The findings demonstrate the serious game framework’s possible applications, including suggestions for combining the various and complementary viewpoints of professionals and young people. Yohka is an Arabic augmented reality storybook application designed for children with ASD and their caregivers to enhance their reading experience and communication. Involving the users in the design process through several iterations helps in promoting usability, especially for this kind of educational and therapeutic technology (Antunes and Madeira 2022). Children with autism usually face different levels of difficulty in communicating and practicing basic life skills, such as reading, due to language delays or intellectual disability, handling written text, and understanding a book with fairy tales. Yohka was designed for people with ASD, with the aim of allowing their caregivers to enhance their reading experience and communication. It shows a more interactive and animated version of the story that helps the child understand it and the interactions between its characters, which makes it enjoyable and therapeutic at the same time. Yohka was created by co-designing experts, students, parents, and therapists/educators, using assistive technologies to improve accessibility and interactive learning with augmented reality technologies such as AR and virtual reality (VR), with many iterations to create an environment that contributes to the education of people with ASD to improve their quality of life. A game design framework (GDF) is suggested by Tsikinas and Xinogalos (2020) to help practitioners, educators, and designers create successful SGs for those with ASD and intellectual disabilities. The suggested GDF has components that should be incorporated into the layout of SGs for individuals with ASD and ID. The three primary axes of the framework are assessment, learning material and game dynamics, and pedagogy. Considering all of these factors is crucial when creating an SG for those with ASD or ID.

An interactive game called Tracking for Autism (TFA) was created especially to encourage more interaction between children with autism. This study suggests an eye-tracking-based framework for creating interactive games for children with autism and a fresh approach to gauge how much their interaction increases as they progress through different levels. The initial exam included conditions such as gazing down, focusing forward, viewing objects, and having dilated pupils. TFA games were demonstrated to be able to raise the degree of interaction among children with autism based on the outcomes of the initial test. The preliminary study’s findings indicated that the children’s interactions increased both before and after they used TFA toys (Azizah et al. 2021).

The authors of Ribeiro Silva et al. (2024) conducted a design experiment to assess the effectiveness of gamified co-design. The technique for guiding the development of educational technology was developed through (i) co-design with experts and (ii) gamification design using DTT (Discrete Trial Training) to approach and assist learning processes. DTT is a psychological therapy that has been demonstrated to be beneficial in children with autism. It is based on responding to stimuli through repetition and reinforcement.

### 4.3. RQ2: What Is the Purpose of Existing Platforms? What Educational Skills Do Serious Game Platforms Improve? A Critical Evaluation of Existing Platforms Aimed at Developing and Improving the Skills of People with ASD

A critical evaluation of the identified articles was performed to assess the advantages, disadvantages, strengths, and weaknesses of existing serious gaming platforms. This involved a comparative analysis of different platforms, taking into account factors such as usability, scalability, and adaptability. By examining the strengths and limitations of these platforms, the analysis sought to provide valuable insights for researchers, developers, and professionals in the field of serious games. This information is vital to inform future advances in platform development and address potential areas of improvement and any problems. Table 2 summarizes the research related to the goal for which the platforms are designed, as well as the suggested educational skills that can be improved using the previously described serious game platform.

Regarding research that focuses on theoretical reviews, Almurashi et al. (2022) evaluated the use of Serious Games (SGs) to improve communication, social, emotional, and behavioral attention deficits in individuals with autism spectrum disorder (ASD). The study utilized technology, augmented reality (AR), and traditional picture-sharing communication systems (PECSs) to create an engaging environment for treatment continuity.

In López-Bouzas and Moral-Pérez (2023), a 70-article study on the use of gamified environments and serious games for people with ASD, the focus is on designing and testing prototypes linked to increasing social and emotional skills. The review highlights that game mechanics and dynamics (feedback, rewards, quests, etc.) engage motivated learners with a positive impact on increasing self-control, self-awareness, autonomy, and empathy in people with ASD. The authors of Camargo et al. (2019), in a review of 53 studies, found that using video games as an assistive tool for ASD intervention resulted in positive improvements in social skills, behavior, gross motor skills, and generalization of acquired skills in the experimental group compared to the control group. In addition, Derks et al. (2022) investigated the relationship between emotional intelligence, games, and learning resources for students with ASD and concluded that a combination of games and emotional intelligence is required for both game design and game research to fully capture the benefits of games for children’s emotional development. In addition, Fridenson-Hayo et al. (2017) supports, in their cross-cultural review (the UK, Israel, and Sweden), Emotiplay’s SG system, which teaches emotion recognition (ER) to children with ASC in a pleasant and stimulating manner. The number of participants in the research in the United Kingdom was 15 children (*n* = 15), 38 children (*n* = 38) in Israel, and 36 children (*n* = 36) in Sweden, all aged 6–9 years with high-functioning ASD and who played the SG for 8–12 weeks. Emotiplay SG was shown to be a successful and stimulating psycho-educational intervention, intercultural teaching tool, and aid for the integration of facial expressions, voices, and body language for children with high-functioning ASC, based on parental feedback. Researchers designed serious purpose games that address the communication domain of people with ASD. The authors of Panceri et al. (2021) created MARIA T21, a “mobile autonomous robot for autistic interaction”, incorporating an SG to enhance psychosocial and cognitive therapy for youth with ASD. As an innovation, the robot has a built-in mini video projector that can project serious games on the floor or tables, providing motivation and convenience for both children and therapists, while serious games examine the theoretical foundations of behavioral psychology for individuals with autism. MARIA T21 is a unique and promising therapy tool for health professionals working with children with autism spectrum disorder and Down syndrome. Because it allows for more engagement, new games may be created to test the child’s capacity to move his/her hands and head, his/her sensitivity to physical contact, his/her ability to portray various facial expressions and emit sounds, and his/her ability to convey emotions. When compared to static toys, the robot can be an effective recreational therapy aid since it encourages higher mind–body involvement and treatment compliance in children with ASD. In addition, Chien et al. (2022) designed a game-based social interaction platform that stimulates gaze tracking, emotional recognition, and social skills. The game-based social interaction platform includes an eye-tracking device for children and adolescents with autism. The platform includes three modules (focused on gaze tracking, facial expression identification, and social interaction skills), which people with autism learn based on their cognitive capacities. The eye-tracking data revealed a shorter fixation length when children with autism looked at positive emotional expressions and focused on numerous objectives, indicating that they are useful biomarkers for assessing the social and cognitive capabilities of persons with autism. The suggested platform is divided into game parts, and research indicates that employing eye-tracking signals in a serious game or real-world scenario would increase the quality of evaluation and intervention processes for children with ASD.

Other researchers created skill platforms for specific circumstances and therapeutic applications. In Antunes and Madeira (2022), game design for treatment sessions enables therapists to share and compare outcomes. A platform concept was created to create a child-centered SG for doctors, therapists, and patients. The technology enables therapists to create tailored SGs that address individual patient requirements. The game model is a versatile tool for game design, allowing for the development of new games that focus on various therapies, resulting in a more thorough therapeutic response.

The platform is well-known as a foundation for ongoing development. New therapeutic activities can be developed and executed, providing a wider spectrum of possible therapies. Increased hardware support for new sensors and actuation devices will result in more involvement in sports and workouts. External applications can be connected to the platform using the offered API (a useful tool for connecting to other programs, platforms, and devices). Research with therapists will determine the model’s validity and platform utility, whereas in Wang et al. (2019), the software promotes emotional detection during therapy sessions. The project’s original mixed-method technique combines ideas from gaming research, design, user experience, psychology, and autism therapy, making it very multidisciplinary. This paper discusses forthcoming research on using electronic games to treat behavioral and neuropsychiatric issues in children with autism, with the goal of improving their emotional awareness. This project will further research gamification in health and well-being, with a focus on providing experiential evaluation for children with autism. The computer game offers an engaging, cost-effective, and low-level intervention solution.

The authors of Luigini and Basso (2021) propose an online application of an immersive serious game as a viable answer to the dilemma of how virtual reality might be applied to distance education. This consists of an internet platform that allows VR apps to be viewed from any device, including on desktop and mobile. The serious game was initially produced on a computer with specific software, utilizing an HMD and using WebXR open-source technologies. The process of turning it into a web platform would allow serious gaming to take place on any sort of platform that can access the Internet. This breakthrough appears to be especially essential in the educational sector, as serious games are used to expose and teach about remote, distant, or fictional places by reproducing them in a digitally accessible setting. Furthermore, the ease of use enables persons with intermediate computer literacy to access the routes, making it suitable for use by instructors or trainers without the assistance of researchers or external professionals. The authors of Zaki et al. (2017) designed and developed an interactive, cost-effective, portable, creative, and user-friendly learning tool that will be exceptionally effective in providing basic academic teaching to persons with ASD. The learning tool includes a pressure-sensitive keyboard, mobility, user-friendly user interfaces (UIs), audio-video support, and a range of interaction possibilities for children with autism. The instrument is evaluated in a laboratory setting to establish its effectiveness and utility. To enhance its usability and technology elements, extensive empirical study will be undertaken with actual users (for example, children with autism) in a real-world context.

Other papers are related to the testing of prototypes. For example, Kirst et al. (2022) examined the effectiveness of an intervention for empathy and emotion detection, using the parent-assisted serious game Zirkus Empathico, which showed some potential for enhancing socio-emotional abilities in children with ASD. Zirkus Empathico is a computer-based educational software designed to promote social-emotional competency in 5- to 10-year-old children with autism spectrum disorders. A six-week multicenter RCT was conducted to compare the serious parent-assisted Zirkus Empathico (ZE) game to an active control group. Eighty-two children with AS aged 5 to 10 years were evaluated at baseline, post-treatment, and three-month follow-up. The major results were empathy and the ability to recognize emotions. Secondary results included assessments of emotional awareness, emotion management, autistic social symptomatology, and subjective treatment objectives. Training effects for empathy and emotion recognition were demonstrated after the intervention but not at the follow-up. Short- and mid-term tests in autism revealed moderate impacts on emotional awareness, emotion management, and social symptomatology. Parents reported successful treatment goals and excellent educational transfer.

The authors of Khowaja and Salim (2019) assess a serious game design framework (SGDF)-based prototype that enables language acquisition using basic graphics. SGs enhance vocabulary learning in children with autism by enhancing their receptive recognition of vocabulary items. An experimental evaluation of the prototype was performed to examine the efficacy of a single-subject withdrawal design (SSRD) research design in boosting receptive recognition of language items among children with autism before and after using the prototype. The number of accurate responses and attempts to obtain the proper answer are used to evaluate receptive recognition of vocabulary words. Pre- and post-evaluations of the SG prototype show that learning vocabulary items in children with ASD improved after playing the game. The objects were kept at the end of weeks 1 and 2 after the intervention was terminated.

For the purposes of their pilot study, the authors of Aresti-Bartolome and Garcia-Zapirain (2015) evaluated a system designed for people with ASD that can help them achieve this goal. The major purpose of the pilot research is to objectively evaluate the interaction between children with ASD and therapists before using this technology as a cognitive rehabilitation aid. The game is designed to be customizable based on the needs of each user and consists of a series of games organized into three levels with increasing degrees of difficulty. At the second and third levels, the game is set to halt every 30 s, either automatically or due to a user error, to measure communication skills and degree of participation with the session leader. When this happens, everything becomes active, and the screen disappears. This stop in the game forces the child with ASD to seek help in order to continue playing, prompting the child to interact with the session leader. The system monitors two types of interactions: (1) the child communicates with the session leader by making eye contact or (2) the child communicates with the session leader using gestures, words, and non-eye-contact exchanges. Saving the interaction is done via the keyboard. Pressing the “space” bar indicates that an eye contact connection has occurred. Pressing the “0” key indicates that a non-eye-contact interaction has occurred. The navigation research found that eye contact dominated the learner–teacher interaction, and the learnt strategy is thought to improve cognitive recovery.

The research focused on the design and subsequent testing in Castillo et al. (2016), the authors of which commenced the AUTHIC project, which is part of assistive technologies and aims to create tools that help children with ASD understand and interpret emotion-related facial expressions through interactive games under the supervision of a clinician. Because research is translational, health findings from the sciences help us comprehend emotion and its universality. Facial expressions enhance multimedia programs built with user-centered and gamification principles. Learning routines allow children with ASD to practice recognizing emotions in an engaging and enjoyable way. This was tested on children with ASD aged between 7 and 15 years old and with a mental age of between 5 and 10 years old. The application was well-received by the patients, since none of the youngsters showed dissatisfaction with their surroundings, but rather enthusiasm for activities. According to the therapists, the children had an excellent understanding of the activities; there was no negative feedback from the children who utilized the platform.

According to Li et al. (2018), a smartphone is a sort of technology that allows individuals to connect with one another while examining their abilities in both social and non-social settings, and it improves executive function (EF), flexibility, and cognition through play in youth. The three game items exhibited a diverse set of EF abilities, including cognitive flexibility (shifting/inference), inhibitory control, and short-term memory. The researchers had 65 children with and without ASD play a mobile game to evaluate the conceptual potential of such platforms in terms of the most common autism-related phenotypic features. The platform includes games for rule switching, short-term memory, and control suspension, and it begins with a game instruction screen that has an audio lecture to teach the user. There are 12 tests in all, separated into three unique mini-games, each with four different activities, to examine various aspects of executive function. The platform captures finger taps (targeted tap data), and when the target is successfully or erroneously tapped, the optimizer generates various sound effects and the target vanishes from the screen, leaving behind a visual particle effect. Research findings demonstrate that play patterns differ between diagnostic groups, with children with ASD exhibiting a wide variety of EF impairments while compensating for increases in nonsocial short-term memory and longer sensitivity to emotional inhibition signals. However, further study is needed to determine the specific nature of social and non-social asymmetry. Creating mobile video games that focus on certain areas of mental health difficulties, with the ultimate objective of developing effective daily monitoring systems for autistic children, is a fantastic concept. There is much research on the growth of socio-emotional abilities. The authors of Barajas et al. (2017) investigate the efficacy of an SG made of natural building blocks similar to Lego and augmented with technology modules to promote therapeutic, social, and cognitive capacities. An SG for children with autism incorporates both a tactile user interface (TUI) and a graphical user interface. The TUI is composed of natural Lego-like construction components that have been enhanced with electrical modules. The suggested SG is meant to be used as a play therapy tool to help children with autism develop their social and cognitive skills. It emphasizes three key skills: intellectual, practical, and social. The preliminary trial results demonstrate that the TUI can improve social interaction and cooperative play in children with ASD. The suggested method improved social contact and collective, game, and exercise performance and reduced lonely game time, indicating that it might be a beneficial tool for play treatment aimed at young children with ASD. In addition, some short-term visual improvements were discovered through feedback during play therapy. The authors of Almeida et al. (2019) created ALTRIRAS, a digital role-playing game (RPG) named after the primary emotions of joy, sadness, anger, and surprise. It was meant to assist literate or illiterate children with ASD aged six to 12 in linking these basic emotions with their associated facial expressions. SG ALTRIRAS is an educational and entertaining game that includes an appealing online environment, entertainment settings with a 2D graphical interface (colors, animations, and other elements), and puzzles to pique children’s interest, as well as an access control and recording system to track the child’s progress. Ten children with ASD and 28 children with neurotypical development (control and experimental groups) completed the game efficacy test. Following the game, all experts and children with neurotypical development filled out the System Usability Scale (SUS) questionnaire. A multidisciplinary team of five experts in each of the following specialties collected data on the functional, non-functional, psychological, and educational requirements, as well as evaluating its consistency and usability. The findings were promising, but children with ASD should spend more time playing to improve their ability to recognize facial emotions.

Furthermore, in the course of treating children with ASD, Armas et al. (2019) developed an enhanced and integrated technological platform that makes use of serious games to boost the emotional and social learning process. The four stages of this platform are data transfer, reporting, analysis, and user registration. The suggested platform makes two significant contributions: first, it looks at the application’s design and the structure of the Participatory Design platform, with the user’s therapist actively participating in every phase of development. Key performance indicators and customized dashboards were developed as a result of user involvement in the design, serving as a business information tool for the therapist. An educational and behavioral therapy center in Peru served as the platform’s testing and validation site. Twenty children aged from three to ten participated in the research. Children were examined before and after SG administration. The app’s revolutionary design allows therapists to collect user data while children interact with play gadgets. To generate individualized therapies, the data are examined by therapists using a mobile app after being transmitted via Wi-Fi or mobile data and saved on a cloud server.

Preliminary results showed a significant improvement in emotion detection after playing the SG, while therapists were particularly happy with the platform’s capabilities. In addition, the platform enables patients from low-income families to participate in group therapy sessions more cheaply using the software on various mobile devices, reducing treatment costs.

The authors of Marchi et al. (2018) describe how an EU-FP7 project will enable children with an autism spectrum condition (ASC), aged 5 to 10, to learn how to express and recognize emotions through play in a virtual environment using facial, vocal, and body gestures. This platform is called ASC-Inclusion Serious Games. The program includes (a) a virtual environment with a theme of a jungle research camp, featuring animated characters and an intelligent incentive system aimed at inspiring children, (b) a learning management application (LMA) that supervises, customizes, and displays instructional materials to parents in order to track their child’s development and conduct and gather pertinent data for further examination and system enhancement, (c) games and exercises, including sophisticated cross-modal games with test games, simple one-mode practice games, and interactive narratives with associated tasks, and (d) 47 interactive courses that explain emotions and provide hints for identifying them through body language, tone of voice, and facial expressions. There is an introductory lesson and a regular lesson for each of the 12 highlighted emotions. The platform’s design is covered in great length in references (Schuller et al. 2013; Schuller et al. 2014; Schuller et al. 2015). Using a built-in microphone and webcam, the platform combines a number of analysis tools that it uses for voice and body movement analysis, educational games, text communication, animations, movies, and audio clips. It also includes a section on formative evaluation and corrective feedback. In addition, the automatic system was modified to accommodate cooperative play for adult individuals and enhanced to meet the clinical teams’ suggestions. The results of the clinical evaluation demonstrated that the platform is a successful educational intervention, as evidenced by the focus group participants’ notable improvement in their ability to recognize emotions and by the clear generalized improvement in socialization and other symptoms common in ASC patients. Future work will concentrate on adding more modes (such as touch) based on motion sensors and different gaming interfaces (Kaplan et al. 2013), dynamically changing the game’s difficulty (Tan et al. 2011), and conducting a more thorough examination of the behavioral play data (Bauckhage et al. 2015).

Additionally, Islam et al. (2022) created an Internet of Things (IoT) platform to enhance cognitive abilities in children with ASD. Three games—a card game, a puzzle game, and a road-crossing game—were created based on the conceptual framework that was created for the platform’s design. The goal of the activities is to enhance the child’s fundamental cognitive abilities, including learning, memory, logic, caution, and attention span. The gaming platform combines a smartphone app with three hardware units. The game was played on a hardware platform, and the player’s progress was shown via a mobile application, where game performance was also controlled, managed, and stored. The device records, stores, and displays progress, as well as the interactions between users (parents, therapists, and children). The gaming platform consists of two primary components: a smartphone app and a hardware game box. The hardware games are controlled by the smartphone app that is connected.

Fifteen youngsters with special needs participated in the evaluation of the proposed platform. The authors of Islam et al. (2022) discovered that the gaming platform really enhanced the cognitive abilities of children on the spectrum and was easy to use, beneficial, and useful. Additionally, the platform was deemed to be fascinating, captivating, and helpful for interactive learning, tracking the development of individuals with ASD over time and comparing things that will enhance their learning and cognitive abilities.

Finally, Vukićević et al. (2019) created four Kinect-based visual motor games called Fruits to help people with ASD receive early motor skills instruction. If any impacts were found, they would be generalized to the game Rackets. The authors of Vukićević et al. (2019) developed a cooperative VR game suite called Fruits to assist children with ASD in developing their motor skills. There are four games in total: Search, Mimic, Sort, and Catch. Every game targets a distinct area of skill development. The Sorting Game offers practice in concentration, visual motor coordination, motor planning, selective attention, and focusing on a specific activity. Catch is a game that tests grip, balance, and head–hand coordination. Playing pretend helps develop gross motor abilities such as jumping, squatting, controlling the head and hands, and maintaining trunk stability. Playing the Search game helps players focus and be creative. All of the previously listed motor abilities must be used in tandem with the augmented reality game Rackets in order to ascertain whether the motor skills learned from the game can be transferred to another game that combines virtual and real-world items. The Fruit and Racket games included a number of strategies linked to designing games for children with ASD, including the methodical use of levels and goals and a system of rewards, avatars, graphics, and analytics. The study’s findings demonstrated that the ten school-age ASD participants, aged 9 to 13, significantly improved their gross motor abilities, successfully generalized newly learned skills, felt happier, and paid less attention to the games they were playing. These early results demonstrate that playing instructional Kinect games improves motor abilities in children with ASD. However, more studies are required to confirm and expand these results with bigger participant populations.

## 5. Results and Discussion

The research design (characteristics, methodology, etc.) of game platforms with serious games was the sole focus of this literature review. Open-access journal articles were included, but neither articles from 2024 nor those referring to platforms with serious games using robots, VR, or other technologies were included.

In Mota et al. (2020), it is highlighted that research on the design of platforms for serious games would benefit from considering the outcomes of earlier tests and guidelines Carvalho et al. (2022) in order to favor and optimize, as well as the design of gamified resources to achieve predetermined goals (Mubin and Poh 2019), their degree of usability (Jaramillo et al. 2022), and other criteria compatible with past research on the creation of settings for the development of serious games for educational interventions for people with ASD. While it is noted that mental health professionals, minors’ hobbies, and designers’ experience are typically not involved in the design of these environments, López-Bouzas and Moral-Pérez (2023) emphasizes that Lee et al. (2020) contends that platforms with SGs do not adjust to the social and health needs of adulthood (work world, emotional relationships, etc.). To create such an environment, it would be beneficial to record and include the perspectives of interdisciplinary teams participating in the education of persons with ASD (Malinverni et al. 2017).

In addition, it is found that the participants of the research are mainly men, while the presence and opinion of the female gender would also be useful (Tang et al. 2019). In addition, as Atherton and Cross (2021) points out, there are few studies supported by large samples as the majority of research has a small sample size. The need for a larger sample size in more research is also pointed out by Hassan et al. (2021) and Silva et al. (2018), who emphasize that a larger sample SG trial should be performed, as well as clinical validation and periodic follow-up.

In addition, due to the heterogeneity of the characteristics of ASD, the environments show an extrapolation of the interventions in multiple contexts Mota et al. (2020) so that they can be used in various ways, facilitating social interactions in various contexts and situations, such as during the pandemic (Elshahawy et al. 2022).

The authors of Fridenson-Hayo et al. (2017) suggest that scenarios can simulate real situations, reducing the cognitive load of identification and internalizing social norms and stimulating language ability. The authors of Khowaja et al. (2018); Elshahawy et al. (2022), and Tang et al. (2019) emphasize the importance of gradually increasing the difficulty of in-game tasks and adapting the game’s demands to the player’s skills.

In addition, Tang et al. (2019) emphasizes the opportunity for SGs to emotionally identify a safe and autonomous environment, becoming a solution to reduce their feelings of frustration and anxiety.

Additionally, Hulusic and Pistoljevic (2017) notes that there are not many suitable SGs in languages other than English for people with ASD.

The in-depth analysis of the collected articles has provided important insights into the field of serious gaming platforms. These platforms offer a rich set of features and tools, allowing developers to create impressive and exciting serious games. Notable features include essentials such as game engines, game editors, and content management systems.

Through the articles used in this work, it was found that some game platforms can be useful tools for people with mild spectrum disorders and provide important information to people with autism spectrum disorder; however, they present advantages and disadvantages, as well as strengths and weak points.

Examining the strengths of existing platforms reveals their remarkable flexibility, hosting a wide range of serious games tailored for various purposes, audiences, and platforms. Additionally, these platforms exhibit commendable scalability, supporting the development of expansive and complex serious games.

In particular, it is important to note the fact that these game platforms, through their flexibility, emphasize user-friendliness and adapt to the needs of people with autism spectrum disorder, helping to improve and develop their skills and abilities.

However, a critical thought arises when considering the weaknesses and disadvantages of these platforms in addition to the advantages. The analysis highlights that cost estimates can emerge as a limiting factor, especially for ambitious and complex projects. Funding the existence of these games involves a significant cost that a parent or educator can hardly afford. Additionally, the complexity of existing serious gaming platforms can pose challenges, particularly for developers with limited experience. People who are not familiar may also find it difficult as it may be quite complex for them.

Addressing design issues in serious gaming systems is crucial. Versatility is key since it allows for adaption to a wide range of serious games. Simultaneously, scalability is emphasized to help in the development of large and complex serious games. Ease of use is emphasized, making it accessible to developers of all levels. As a result, it is necessary to solve the challenges posed by serious gaming platforms that provide guidance for simple modification and flexibility to persons with ASD. In order to ensure that these platforms are accessible to everyone, they must be simple to use.

Therefore, it is important to note that the platform faces some strengths as well as some weaknesses and challenges. The strengths include the flexibility and scalability of these games, while the weaknesses include the complexity of the platforms as well as their increased cost.

The authors of Grossard et al. (2017) investigated and evaluated social game design aspects that increase social contact among people with ASD in a 31-article analysis of serious games published between January 2001 and April 2014. The activities attempt to improve social skills. Sixteen of these games attempted to identify or create facial emotions, “although the social skills required in real life include rich combinations of perspective taking, emotional regulation, cognitive flexibility, and appropriate use of language”. The game design has concentrated on the core capacity of emotion perception, which enables more advanced types of social skills (Chaidi and Drigas 2020).

The authors of Grossard et al. (2017) investigated the existence or absence of the numerous serious gaming criteria proposed by Yusoff (2010). They also looked into SGs that encourage social engagement amongst people with ASD and proposed design guidelines. The games typically have numerous components. However, three qualities were less commonly used: attention span, motivation, and student hospitality. Each game was unique, with adjustments based on the writers’ selections.

Research is needed to identify whether the potential of these resources is sustained over time or just utilized during the intervention. Regarding this, Grossard et al. (2019) discovered that, while there are games for identifying facial expressions, few focus on developing contextually appropriate expressions (López-Bouzas and Moral-Pérez 2023).

According to López-Bouzas and Moral-Pérez (2023), these resources have a positive impact on self-control, self-awareness, autonomy, and empathy. Additionally, the game’s mechanics and dynamics, such as feedback, rewards, and quests, motivate students.

The authors of Grossard et al. (2017) suggest that future research should focus on developing gamified and SG environments for high-functioning individuals (Fridenson-Hayo et al. 2017; Terlouw et al. 2021). As a result, it will be required to create venues for meaningful study that will reach a broader range of people on the autism spectrum. As López-Bouzas and Moral-Pérez (2023) mentions, clinical validation of gamified or social gaming settings may not always be compatible with replay control (Hulusic and Pistoljevic 2017). The goal of this research is to determine whether the potential of these resources is sustained over time or is only documented at the point of intervention (Grossard et al. 2019; Terlouw et al. 2021).

It should be emphasized that the cost of developing high-quality serious game platforms is expensive, as they require specialized knowledge in both education and game design.

In addition, access for people with ASD is an issue. Not all students may have access to the devices or Internet connections needed to play serious digital games, potentially widening the digital divide.

Finally, an overemphasis on fun may be detrimental. While engagement is critical, a serious game that prioritizes fun over educational content can fail.

Summarizing the strengths, existing serious gaming platforms exhibit commendable flexibility and scalability, purposefully designed to serve developers of varying skill levels. However, challenges may arise in the form of cost and complexity considerations, which require careful consideration when selecting a platform for specific projects. Essentially, the analysis highlights the central role of serious gaming platforms’ ineffective game development, emphasizing the importance of a nuanced approach and consideration of design elements, strengths, and weaknesses in platform selection.

## Figures and Tables

**Table 1:** Studies on Platform Design for Individuals with ASD. Source: own elaboration.

Studies by Platform Design for Individuals with ASD				
Authorship (Date) Nationality	Design of the Research			
	Method	Population	Aims	Procedure
Khowaja and Salim (2014) Malaysia	Design	Children with ASD.	Identifying evidence of their choice in the application of creating serious games for children with ASD.	The framework elements that designers and professionals need to create serious games for children with ASD.
Whyte et al. (2014) USA	Design	Individuals with ASD.	Interventions with serious games should consider the full range of serious game design principles that promote its generalization learning.	Basic principles of serious game design and consideration of the use of these principles in computer-based interventions for people with autism.
Bono et al. (2016) GOLIAH Italy	Principles And Design	10 children with ASD.	Feasibility of using the developed gaming platform for home-based intensive intervention.	An automated serious gaming platform for intensive intervention in nomadic settings has been created by mapping two essential skills in ASD: imitation and joint attention (JA).
Tuli and Mantri (2018) India	Co-Design Using the proposed game development process, we developed two serious games	30 students with ASD in the 8th and 10th classes.	The research aims to present a model for the design and development of educational games with the integration of pedagogical and design elements and the distribution of roles between the players in the development process.	The paper presents some of the important issues faced during development that need to be analyzed to support research in SG development.
Alarcon-Licona et al. (2018), Australia	Design	A primary school for children with ASD, 15 students (aged 5–12).	The goal of this project was to create games and interactive artifacts to assist children with ASD in dealing with sensory problems at school.	A game design technique was created to enhance autism education.
Tang et al. (2019). Australia	Design	11 young people with ASD (*n* = 11, one female) aged between 13 and 24. 11 professionals (*n* = 11, 5 women). 22 CBI stakeholders with the aim of improving sentiment recognition skills in young people with ASD.	This study aimed to obtain suggestions from youth with ASD and professionals on methods to practically apply the five serious game principles.	This study reveals critical motivational and learning aspects of serious games that emphasize emotion recognition skills, as seen by 11 children with ASD and 11 experienced professionals.
Camargo et al. (2019) Brazil	Systematic Review	People with ASD.	The purpose of this article is to illustrate a wide range of gamification components, strategies, and approaches for improving accessibility and assisting decision-making in the creation of autism-specific software.	This systematic study looks at the present state of gamification tools for autism, focusing on the gamification components and user interface design.
Alkadhi et al. (2020) Saudi Arabia	Design	As an initial step and until the team obtains access to children with autism, the application was tested on two typically developing children, aged 5 and 7 years old.	The goal is to shed light on the prior state-of-the-art in this subject and identify opportunities for further study and development.	Yohka is an Arabic augmented reality book app designed to improve communication and reading skills for children with ASD and their caregivers.
Tsikinas and Xinogalos (2020). Greece	Design	Students and young adults with ID and ASD.	This work intends to provide a specialized and informed GDF for creating SGs for individuals with ID and ASD, addressing a need in the field.	This article proposes a serious games design framework (GDF) to help designers, special education instructors, and professionals create effective SGs to develop and improve social skills for individuals with intellectual disabilities and ASD.
Atherton and Cross (2021) USA	Literature Review	Individuals with Autism	This overview is aimed at educators, professionals, and parents of people with autism. It focuses on how research on gamification and autism can progress and be applied.	It reviews studies that have tested game-based approaches to improve the lives of autistic children, adolescents, and adults.
Azizah et al. (2021) Indonesia	Designing interactive games for autistic children based on eye tracking	Children with autism.	The aim of the thesis is to present an eye-tracking-based framework for creating interactive games for children with autism, as well as a new technique for determining the increase in the interactions of children with autism from one level to another.	A new game design based on Tracking for Autism (TFA) to improve the interaction of children with autism.
Ribeiro Silva et al. (2024). Brazil	Co-Design and testing	4 children with autism, aged 7 to 12-years-old.	The original DTT (Discrete Trial Training) application was used as an approach to support the learning activities of people with autism.	A design experiment to determine the efficacy of gamified co-design.

**Table 2:** Studies by serious gaming platforms for Individuals with Autism. Source: own elaboration.

Studies by Serious Gaming Platforms for Individuals with Autism				
Authorship (Date) Nationality	Design of the Research			
	Method	Population	Aims	Procedure
Aresti-Bartolome and Garcia-Zapirain (2015). Spain	Testing	A group of 20 children diagnosed with ASD aged between 3 and 8 years old (clinical group). A group of 20 children aged between 3 and 8 years old with neurotypical development.	Assess the usefulness (using eye-tracking metrics) of serious games to include them as a cognitive rehabilitation aid.	A touch screen and tactile pointer-based serious game app.
Castillo et al. (2016). Mexico	Testing and design	Children aged between 7 and 15 years old with ASD and a mental age of between 5 and 10 years old.	Teaching and identifying fundamental emotions.	Web environment.
Barajas et al. (2017). Canada	Design and testing.	6 children with ASD, aged up to 9 years, in two teams.	Create a tool that improves social and cognitive abilities.	Testing of an SG supported by lego blocks.
Zaki et al. (2017). Bagladeshh	Design		Teach fundamental academics (English alphabet) utilizing a pressure-sensitive keypad.	Portable learning tool.
Fridenson-Hayo et al. (2017). UK, Israel, and Sweden	Review	Participants were 6–9 years old with high-functioning ASC who used the SG for 8–12 weeks. In the UK, 15 children. In Israel, (*n* = 38) and in Sweden, (*n* = 36).	Cross-cultural efficacy review of a serious game to teach awareness of emotions (using the face, voice, body, and their integration) to children with autism.	Serious game-based online Emotiplay app.
Li et al. (2018). USA	Design and testing	65 children with and without ASD	Improve executive function (fexibility and cognitive function) through play.	Design and testing of a mobile game that uses social stimuli with 65 subjects.
Vukićević et al. (2019). Serbia	Design and testing	The study included 10 elementary school children with ASD, aged 9–13 years.	The aim is to improve the implementation of early motor skills intervention with behavioral changes during game use.	As educational games and modern technology can represent new forms of treatment, this study evaluated four Kinect-based visual–motor games called Fruits that were specially designed for this research.
Marchi et al. (2018). Germany	Design and testing	The first clinical trial was conducted in the UK. Fifteen children with an ASC. A selection of children from Israel and Sweden (38 and 36, respectively), aged 6–9 years.	The ASC-Inclusion EU-FP7-funded project aims to provide children who have an ASC with a platform to learn emotion expression and recognition through play in the virtual world.	Τhe perceptual serious game platform ASC-Inclusion, designed for children with an ASC aged from 5 to 10 years. The ASC-Inclusion platform focuses on the expression of emotion via facial, vocal, and bodily gestures.
Almeida et al. (2019). Brazil	Design and testing	10 children with ASD and 28 children with neurotypical development	Recognize facial expressions associated with the four basic emotions: joy, sadness, anger, and surprise.	A computer game, ALTRIRAS, developed to assist children with ASD.
Khowaja et al. (2018). Malaysia	Testing	5 children with autism	Testing an SG prototype which helps in vocabulary learning.	Survey to teachers, prototype design, and Intervention.
Armas et al. (2019). Peru	Design and testing.	20 children between ages of 3 to 10 years old participated in the study.	Optimize the process of emotional and social learning therapy in treating children with ASD.	Proposed an enhanced and a comprehensive technological platform using serious games.
Arzone et al. (2020). Malaysia	Review	Fifteen pupils with ASD were directly involved in this study.	Study the role of gamified environments to increase emotional intelligence (EI).	Literature review to establish guidelines for designing environments to improve EI.
Silva et al. (2021). Brazil	A Systematic Review	Individuals with ASD	The use of serious games and entertainment games was compared as adjuvant tools for intervention in ASD.	53 studies were selected and included in this review.
Luigini and Basso (2021). Italy	Review		A web-based application of an immersive serious game was proposed as an appropriate method for addressing the challenge of how virtual reality (VR) may be applied to distant learning.	Web-based serious game.
Panceri et al. (2021). Brazil	Design	8 children (one child with typical development, one with Trisomy 21, both female, and six children with ASD (one girl and five boys), from 4 to 9 years of age.	Improve psychosocial therapies.	Development of a robot that integrates SGs.
Wang et al. (2019). China	Design	Individuals with ASD.	Stimulate emotional understanding.	Software design to facilitate therapies.
Almurashi et al. (2022). Saudi Arabia	Review Literature	Individuals with ASD.	Ρeview on augmented reality, serious games, and PECS.	Search and comparison of 55 studies.
Chien et al. (2022). Taiwan	Design		Stimulate gaze tracking, emotional recognition, and social interaction.	Design of a social interaction platform based on an SG.
Antunes and Madeira (2022). Portugal	Design	Individuals with SN.	Improve the commitment and motivation of patients in therapeutic sessions.	Platform design that allows creating games applicable to specific therapies.
Islam et al. (2022) Bangladesh	Design and testing	15 children with special needs. Their average age was approximately 5 years and ranged between four and 9 years.	The platform attempts to help youngsters develop their cognitive skills.	This article aims to develop, create, and evaluate an IoT-based serious gaming platform for children with ASD.
Kirst et al. (2022) Germany	Testing	82 children aged 5–10 years with ASD.	The aim of the research is the results of the intervention for empathy and the recognition of emotions.	Zirkus Empathico serious parent-assisted play has some potential for training social-emotional skills in children on the autism spectrum.
López-Bouzas and Moral-Pérez (2023) Spain	Review of Research	Students with Autistic Spectrum Disorder.	The aim is to review on the use of Gamified Environments and Serious Games for people with SD, focusing on designing and testing prototypes linked to increasing social and emotional skills.	Τhis study reviews 70 articles.
